# Engineering Axl specific CAR and SynNotch receptor for cancer therapy

**DOI:** 10.1038/s41598-018-22252-6

**Published:** 2018-03-01

**Authors:** Jang Hwan Cho, Atsushi Okuma, Dalal Al-Rubaye, Ejaj Intisar, Richard P. Junghans, Wilson W. Wong

**Affiliations:** 1 Department of Biomedical Engineering, Boston University, Boston, MA 02215 USA; 2 Biological Design Center, Boston University, Boston, MA 02215 USA; 3 Biotechnology Department, College of Science, University of Baghdad, Baghdad, Iraq; 4 School of Medicine, Tufts University, Boston, MA 02111 USA

**Keywords:** Immunotherapy, Synthetic biology

## Abstract

Axl is a tyrosine kinase receptor that is commonly overexpressed in many cancers. As such, Axl represents an attractive therapeutic target. The transfer of engineered T cell expressing chimeric antigen receptor (CAR) is an exciting cancer therapeutic approach that shows high efficacy against cancers in clinical trials, especially for B cell malignancies. Furthermore, recently developed synthetic Notch (synNotch) receptor has demonstrated potential in enhancing the specificity of CAR T cell therapy and delivering therapeutic payloads to tumors in an antigen-dependent manner. Therefore, a CAR or synNotch against Axl could be a valuable therapeutic reagent against many cancers. Here, we develop a single-chain variable fragment from a humanized monoclonal antibody against Axl. The scFv is attached to CD3ζ, CD28, and 4-1BB signaling domains to generate an anti-Axl CAR. When introduced into human primary T cells, the anti-Axl CAR can lead to cytokine production and cell killing in response to tumor cells expressing Axl. Moreover, an anti-Axl synNotch generated using the same scFv can be activated with Axl expressing tumor cells. Given the fact that Axl is an important cancer therapeutic target, these receptors could be valuable reagents for developing anti-Axl therapies.

## Introduction

Receptor tyrosine kinases (RTKs) are transmembrane proteins that sense extracellular ligands. Ligand engagement induces receptor dimerization, which leads to activation of downstream signaling pathways. RTKs regulate a wide range of cellular processes such as cell survival, growth, and differentiation. Moreover, mutation or dysregulation of RTKs has been implicated in many diseases including cancer^1,2^.

The Axl protein is a member of the TAM (TYRO3, AXL, and MER) of receptor tyrosine kinases subfamily and involved in the stimulation of cell proliferation^3^. The Axl receptor has been demonstrated to be overexpressed in many human cancers including breast, lung, colon, and pancreatic cancers. High level of Axl expression is associated with poor prognosis in different types of cancer^4–7^. Oncogenic Axl signaling increases cancer cell survival, migration, and invasion^8^. Dysregulation of Axl signaling is also known to enhance the epithelial-mesenchymal transition (EMT) and cause drug resistance to immunotherapy and chemotherapy^9–12^. Since Axl is implicated in many cancer progression and drug resistance, a therapeutic that targets Axl could be a valuable cancer therapy. As such, antibody, small molecule inhibitors, and Axl receptor decoy are in the preclinical and clinical stage for breast, lung for other advanced solid tumors^13^.

The transfer of tumor-targeting T cells to patients is a promising approach for cancer immunotherapy. In such approach, T cells are isolated from the patient, and tumor-specific receptors such as chimeric antigen receptors (CARs) are introduced into the T cells to redirect their specificity. CARs are composed of an antigen-specific scFv and intracellular signaling domains (CD3ζ and costimulatory domains). The binding of scFv to an antigen on cancer cells will stimulate T cell receptor and costimulatory pathways, leading to the activation of T cells. CAR-expressing T cells have demonstrated unprecedented efficacy against acute lymphoblastic leukemia (ALL), with around 90% complete remission being observed in clinical trials^14–17^. Despite these encouraging results and the recent FDA approval of anti-CD19 CAR T cells for ALL and lymphoma, more antigen-specific CARs are needed to treat cancer beyond B-cell malignancies. Therefore, the development of CAR against Axl could expand the therapeutic range of CAR T cell therapy.

More recently, Lim and colleagues have created a novel receptor design called synNotch that enables the programming of both input and output via the release of intracellular transcription factor upon antigen-receptor binding^18,19^. Unlike conventional CAR activation which triggers endogenous T cell receptor signaling pathway^20,21^, synNotch receptor uses the regulatory notch core portion with an engineered transcription factor that enables programmable inputs and outputs to perform user-defined functions. Because of high programmability, synNotch has been used to reprogram human primary T cell responses both *in vitro* and *in vivo* for enhancing tumor specificity and delivering therapeutic payloads in a tumor antigen-specific manner. As such, synNotch receptor targeting Axl ligand with different output functions, such as producing a defined set of cytokines, will improve cellular immunotherapy to treat various cancers. In this study, we designed a humanized single chain variable fragment (scFv) against Axl. Using our Axl scFv, we engineered an Axl CAR and Axl synNotch receptors. In an *in vivo* setting, we demonstrated Axl CAR in human primary T cells for killing tumor cells and Axl SynNotch receptor for producing IL-10 in an antigen-specific manner.

## Results

### Design and characterization of the humanized Axl CAR

**Figure 1:**
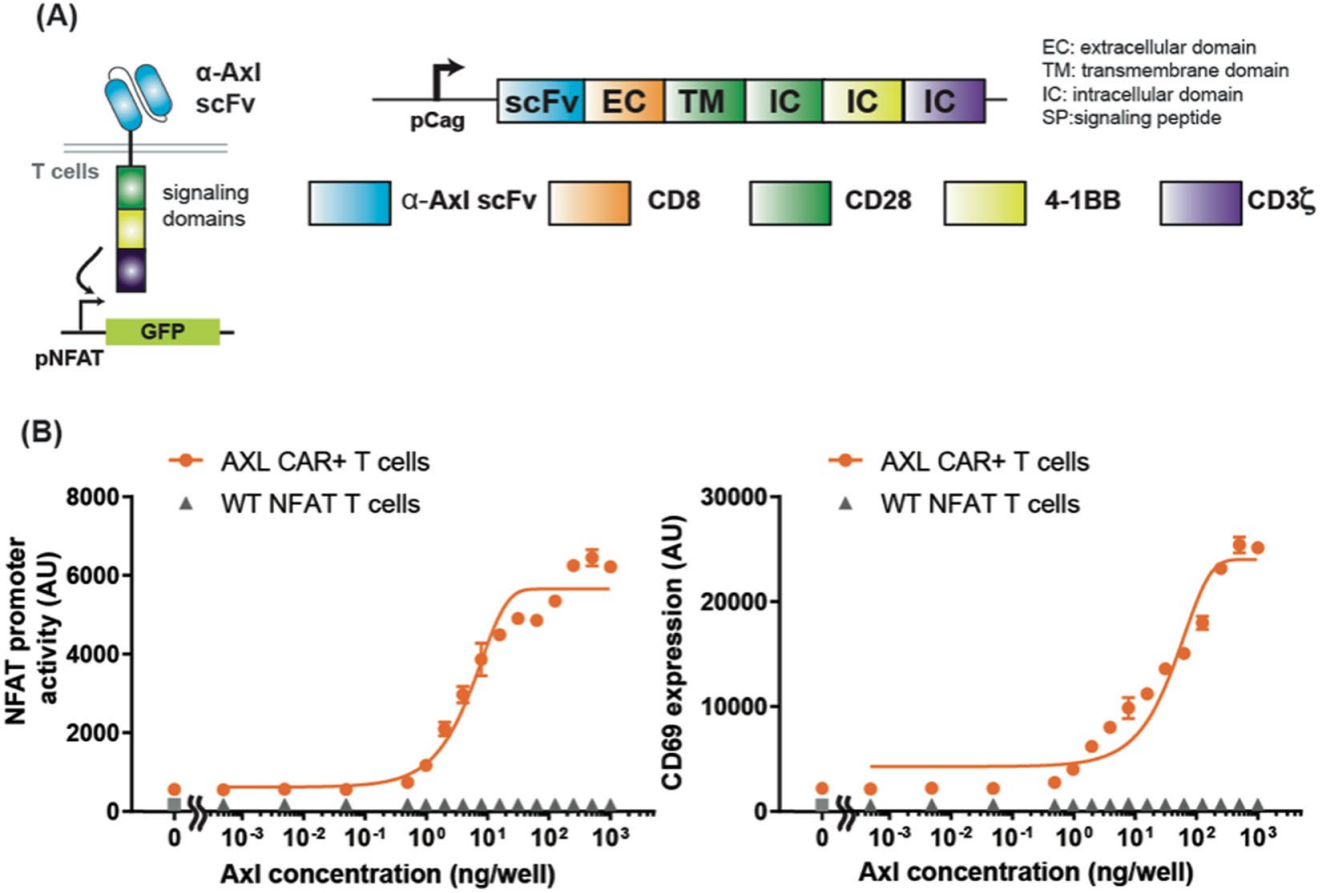
Design and characterization of the Axl CAR. (**A**) Humanized Axl CAR is composed of a humanized Axl scFv as the extracellular domain and CD28, 4-1BB, and CD3ζ signaling domain as the intracellular domain. (**B**) The NFAT promoter activity and CD69 expression levels of Axl CAR-expressing Jurkat T cells after 24 hr of culturing with different amount of plate-bound Axl protein. WT NFAT T cells indicate Jurkat T cells harboring an NFAT reporter without the Axl CAR. Data are representative of three biological replicates and presented as the mean ± standard deviation (SD).

Since the receptor tyrosine kinase, Axl, is overexpressed in many different types of cancer, we tested if we can design a humanized single chain variable fragment (scFv) against Axl that can be used for cellular immunotherapy, especially in the context of CAR and synNotch receptor. From a previously published humanized Axl antibody sequence, we designed an Axl scFv by fusing a variable region of heavy chain to light chain through a GS linker^4,22^. We first tested the functionality of the Axl scFv by using it to create an Axl CAR. The Axl CAR is comprised of the Axl scFv and CD8α hinge region as the extracellular domain, and CD28, 4–1BB, and CD3ζ as the intracellular signaling domains (3^rd^ generation CAR^20,23^)(Fig. 1A). To verify the activity of the Axl CAR, we stably integrated Axl CAR in Jurkat T cells genome through the electroporation of the PiggyBac transposon system^24^. This Jurkat T cell line also contains an NFAT promoter driving GFP expression for measuring CAR activation. As NFAT is a representative transcriptional factor that is known to be activated after T cell receptor (TCR) activation^23^. Therefore, NFAT transcription response is used to measure T cell activation by Axl CAR. After Axl CAR-expressing Jurkat T cells were stimulated with plate-bound Axl protein, Axl CAR-expressing Jurkat T cells displayed a high level of CD69, which is an early T cell surface activation marker^25^, and NFAT transcription reporter activity measured by GFP expression (Fig. 1B). In contrast, Jurkat T cells without Axl CAR did not yield high CD69 and NFAT reporter expression.

**Figure 2:**
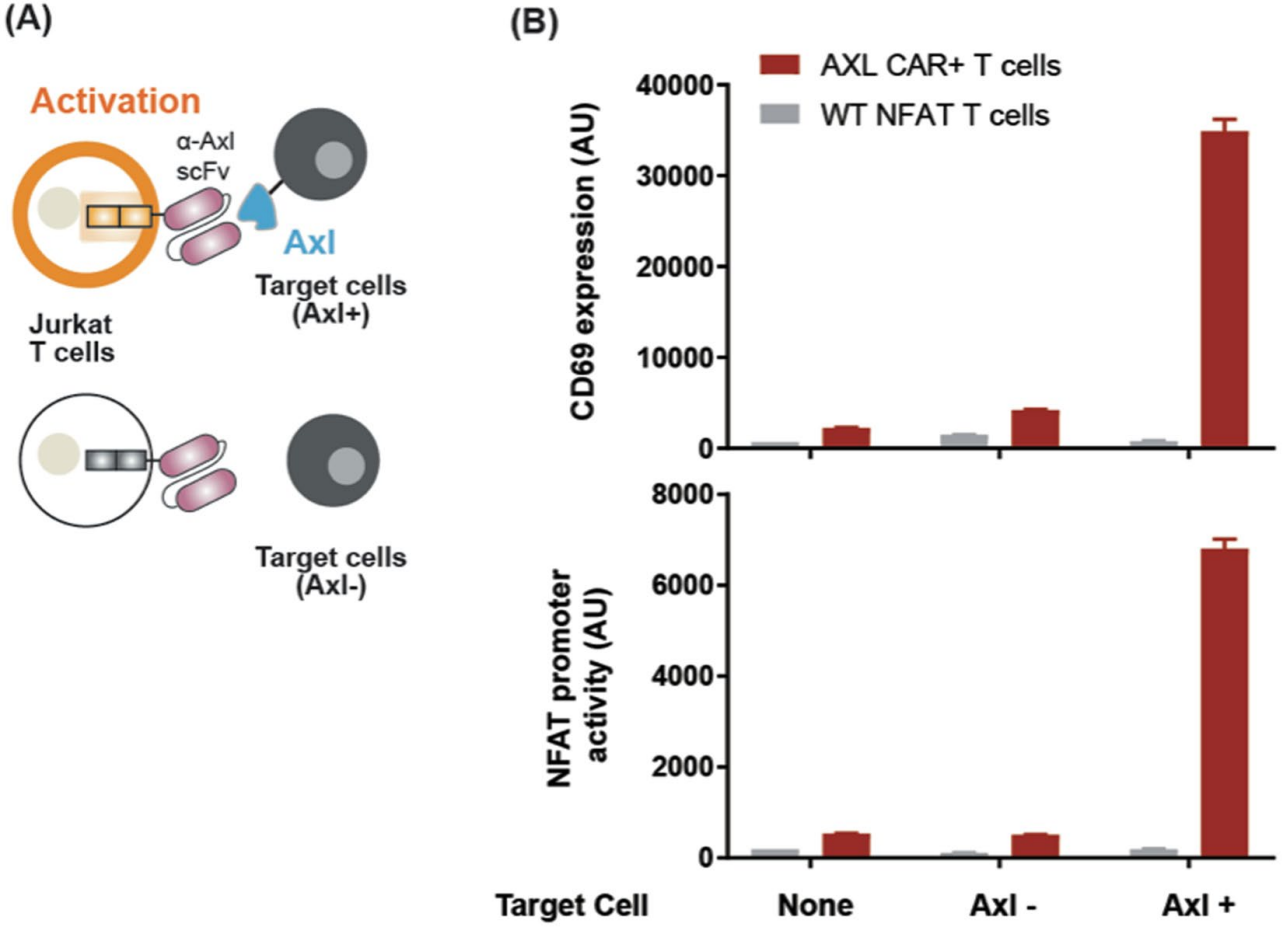
Axl CAR activation via cell-cell interaction. (**A**) Axl CAR-expressing or wild-type NFAT Jurkat T cells were co-cultured *in vitro* with Axl+ or Axl− K562 cells. (**B**) The NFAT promoter activity and CD69 expression level were measured after Axl CAR-expressing Jurkat T cells, and Axl+ K562 cells were co-cultured for 24 hr. Data are representative of three biological replicates and presented as the mean ± SD.

To test Axl CAR activation under a more physiologically relevant condition, we engineered K562 myelogenous leukemia cells to express the Axl antigen. Axl CAR-expressing Jurkat T cells were then co-cultured *in vitro* with Axl+ K562 cells (Fig. 2A). Axl+ K562 cells activated Axl CAR-expressing Jurkat T cells strongly as measured with CD69 and NFAT transcription reporter expression. However, Axl CAR T cells were not activated by Axl− K562 cells (Fig. 2B). Furthermore, basal activity of Axl CAR was minimal as measured by both CD69 and NFAT transcription reporter expression (Fig. 2B).

### Characterization of Axl CAR in human primary CD8+ T cells

**Figure 3:**
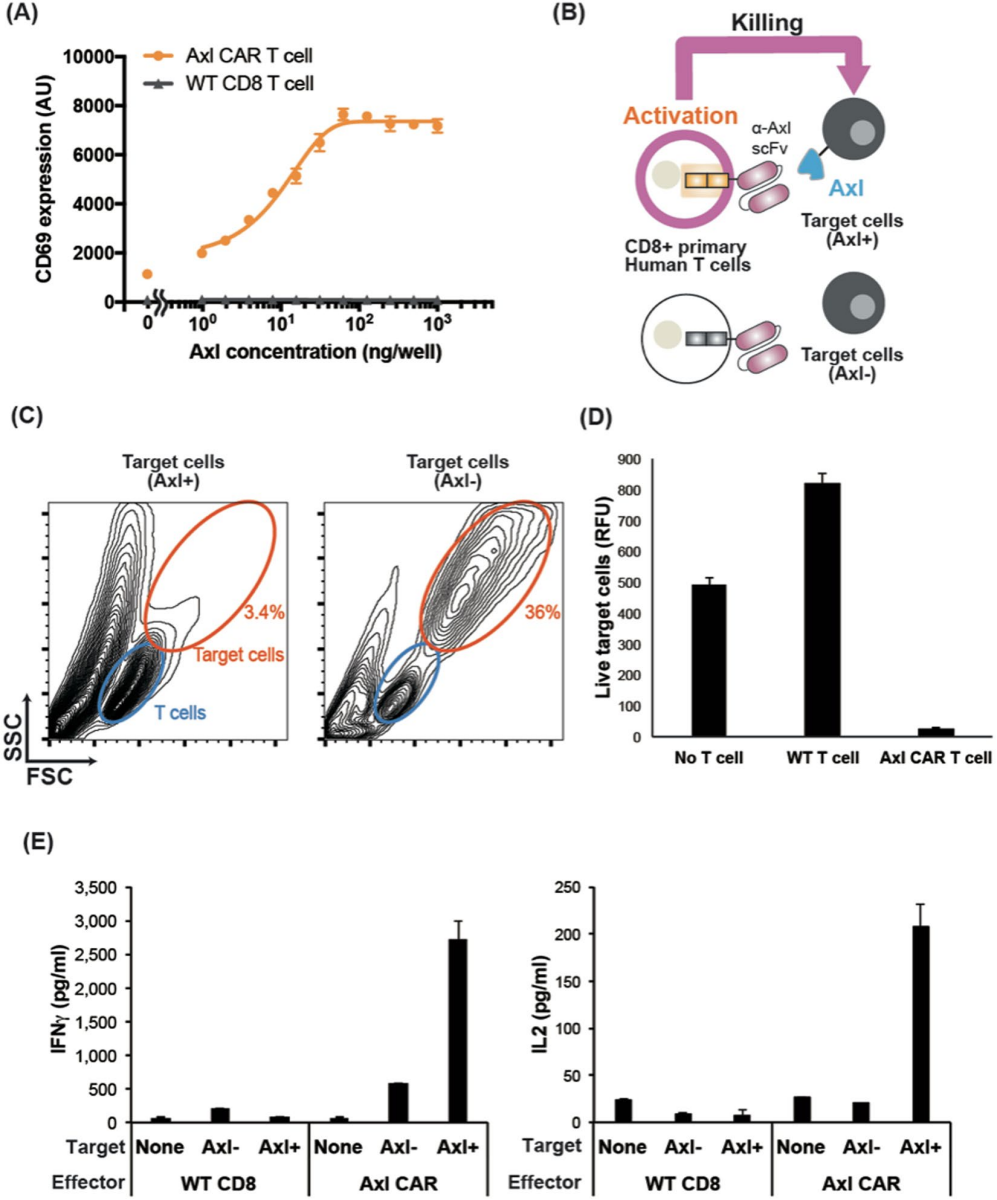
Characterization of Axl CAR in human primary CD8+ T cells. (**A**) The CD69 expression level measured after 24 hr of culturing Axl CAR-expressing CD8+ T cell with a different amount of plate-bound Axl protein. (**B**) Schematics of cell killing against K562 target cells by Axl CAR-expressing CD8+ T cells. (**C**) Forward- and side- scatter FACS plots of the cell mixture after 24 hr co-culture of T cells (blue) with target cells (orange). (**D**) Killing assay against SK-OV-3. Fluorescence of Calcein AM was used to quantify live SK-OV3 cells after 24 hr co-culture with T cells. (**E**) IFN-γ and IL-2 measurement after 24 hr co-culture of human primary CD8+ T cells with Axl expressing target cells (K562). Data are representative of three biological replicates and presented as the mean ± SD.

After characterizing Axl CAR in Jurkat T cells, we tested whether our Axl CAR is active in human primary T cells. Human primary CD8+ T cells were engineered to express the Axl CAR through lentiviral transduction, and we verified via flow cytometry analysis that more than 80% of the cells expressed the Axl CAR (Supplementary Fig. 1A). To determine whether the Axl CAR is functional in CD8+ T cells, the engineered T cells were activated with plate-bound Axl. High CD69 expression on the T cells confirmed that Axl CAR T cells could be activated from plate-bound Axl protein (Fig. 3A). Next, we tested if engineered T cells can eliminate Axl+ tumor cells. We co-cultured Axl CAR T cells with target cells (Axl− or Axl+ K562) (Fig. 3B). Forward- and side- scatter FACS plots of the cell mixture after 24 hours co-culture of T cells with K562 tumor cell showed that Axl CAR-expressing CD8+ T cells could kill Axl+ tumor cells efficiently (Fig. 3C left). However, Axl CAR-expressing T cells did not kill Axl negative target cells (Fig. 3C right). Consistent with this result, live K526 cell counts, which were gated by 7-AAD-negative and fluorescent markers, decreased only in the co-culture of Axl CAR T cells with Axl+ K562 cells (Supplementary Fig. 1B). Furthermore, we tested the killing efficiency against Axl expressing Jurkat T cells, and Jurkat T cells were killed by Axl CAR-expressing CD8+ T cells in a dose-dependent manner (Supplementary Fig. 1C). Importantly, Axl CAR T cells also killed endogenous Axl-expressing tumor cells, such as SK-OV-3 an ovarian cancer cell line, demonstrating the clinical relevance of Axl CAR (Fig. 3D). We next tested cytokine secretion and verified that Axl CAR-expressing human primary CD8+ T cells secreted high level of IFN-γ and IL-2 only when they were co-cultured with Axl+ K562 tumor cells (Fig. 3E).

### Design and characterization of the humanized Axl synNotch

**Figure 4:**
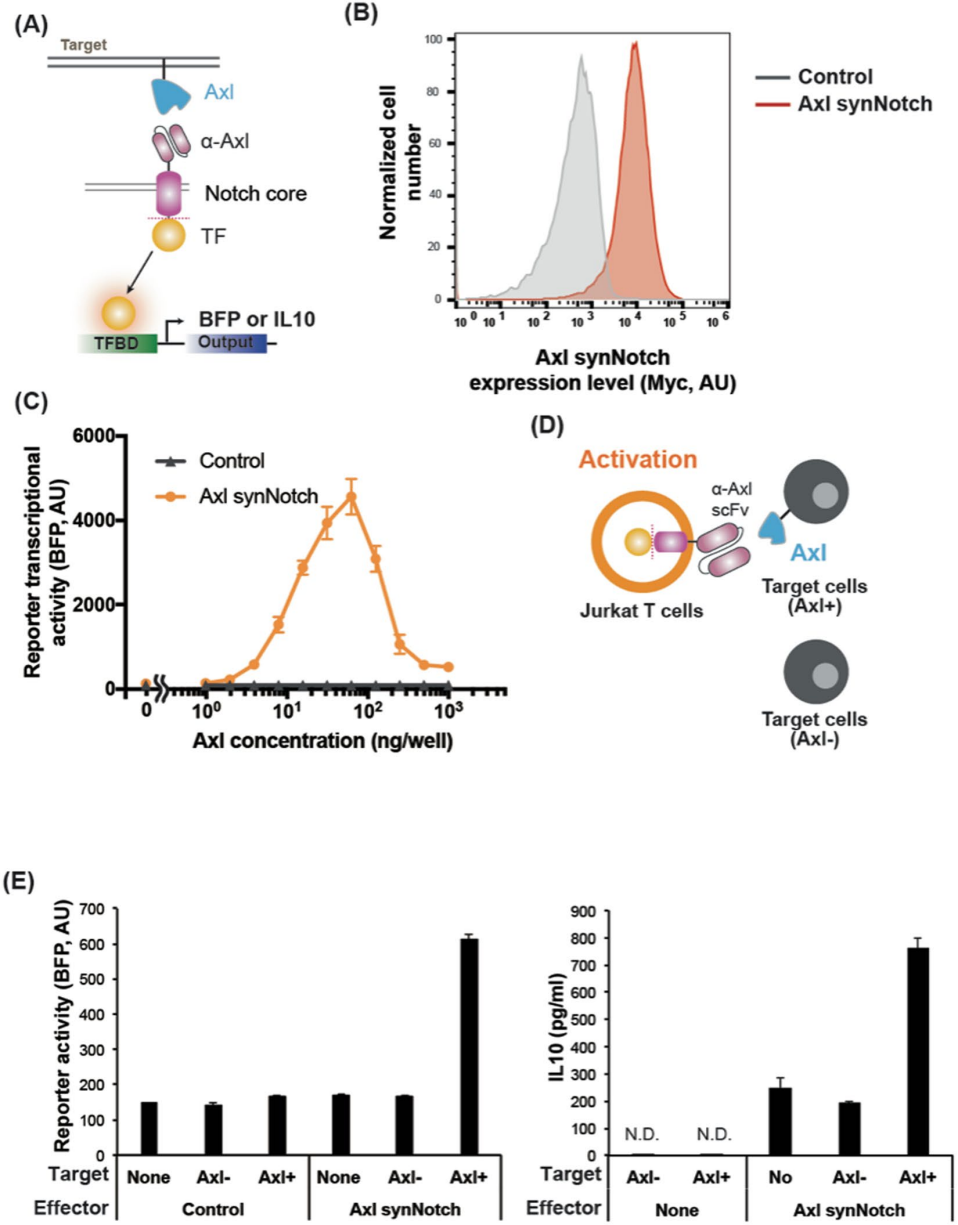
Design and characterization of Axl synNotch in human Jurkat T cells. (**A**) Axl synNotch design. TF, transcriptional factor (tTA); TFBD, transcriptional factor binding domain. (**B**) The expression level of the Axl synNotch in Jurkat T cells. The myc-tag was stained with an anti-myc antibody for the measurement of surface synNotch expression. Control indicates non-transfected Jurkat cells that containing only tTA responsive reporter. (**C**) Axl synNotch response from plate bound Axl protein activation. Control indicates Jurkat T cells harboring synNotch responsible BFP reporter without Axl synNotch. (**D**) Axl synNotch activation via co-culturing of Axl+/Axl− K562 target cells. (**E**) BFP fluorescence level after co-culturing of Axl synNotch expressing Jurkat T cells with target K562 cells for 24 hr. Control indicates Jurkat T cells harboring only BFP reporter without Axl synNotch receptor. (**F**) IL-10 production level when co-cultured Jurkat T cells harboring tTA responsive IL-10 reporter cells with K562 cells for 24 hr. (None, no target cell or no effector cell condition. N.D., not detected: Data are representative of three biological replicates and presented as the mean ± SD).

Recently, Lim and colleague demonstrated the use of synNotch receptors for cellular immunotherapy applications^18,19^. Here, we tested if the Axl scFv can be utilized to generate a functional synNotch receptor. The Axl synNotch receptor is composed of the Axl scFv as the extracellular domain and notch core region fused to engineered transcription factor (tTA) (Fig. 4A). Jurkat T cells were engineered to stably express Axl synNotch receptor using electroporation and piggyBac transposon-based system. The expression of the Axl SynNotch was verified by α-myc cell surface staining with flow cytometry (Fig. 4B). A reporter construct that composes of a tTA responsive promoter followed by a gene of interest (e.g., blue fluorescent protein (BFP) or IL-10) were also introduced into Jurkat T cells^18,19,26^. IL-10 was chosen as an output because lL-10 is an inhibitory cytokine that can be used to control inflammation^18^. When Axl synNotch expressing T cells engage with tumor cells that express Axl, the tTA transcription factor is cleaved from the synNotch and translocated into the nucleus to regulate gene expression from the reporter.

To test the functionality of the Axl synNotch receptor, Axl synNotch-expressing Jurkat T cells were stimulated with plate-bound Axl protein, and BFP expression from the synNotch transcription reporter was quantified. Interestingly, the dose-response curve of the Axl synNotch receptor displayed a Bell curve characteristics, with maximum activation occurring at ~100ng of Axl protein (Fig. 4C). We also tested if Axl synNotch receptor could be activated with tumor cells that express Axl (Fig. 4D). Jurkat T cells expressing the Axl synNotch were activated and produced BFP only when co-cultured with Axl+ K562 tumor cells for 24 hr (Fig. 4E), whereas cells containing tTA responsive element without the Axl SynNotch did not have high BFP expression (control, Fig. 4E) even when co-cultured with Axl+ tumor cells. Consistently, Jurkat T cells that were transiently transfected with tTA responsive IL-10 reporter showed a similar result, activating and secreting IL-10 only when co-cultured with Axl+ tumor cells (Fig. 4F). Note that the basal activity of Axl synNotch is minimal when compared to Axl− K562 cell condition and no target cell condition. These results demonstrate the potential cellular immunotherapy application using Axl synNotch receptor.

## Discussion

Genetic engineering of T cell for cellular immunotherapy application has become a promising cancer therapeutic approach. While multiple clinical trials against B cell malignancies have shown encouraging results, CARs that target antigens overexpressed in other cancers besides B cell tumors are still needed. In addition to CARs, the recently developed synNotch receptor have displayed novel therapeutic capabilities by enabling programmable T cell responses such as user-defined cytokine secretion, T cell differentiation, and local delivery of therapeutic antibodies. However, only anti-CD19, GFP, or Her2 synNotch receptors have been explored. In this study, we developed a humanized Axl scFv from previously reported Axl monoclonal antibody by fusing variable region of heavy and light chain via a polypeptide linker. Using this Axl scFv, we successfully created the first Axl CAR and synNotch receptor, which can be valuable therapeutic reagents since Axl is overexpressed in many cancers including colon, breast, prostate, pancreatic and lung cancers.

The anti-Axl CAR and synNotch behaved mostly as expected. To demonstrate the clinical potential of our Axl receptors, further studies in animal models will be required. Interestingly, we demonstrated that Axl synNotch receptor can be strongly activated by plate-bound Axl protein and observed that optimal activation of Axl synNotch receptor occurred when Axl protein was plated at 100ng/well. Moreover, we showed that Axl synNotch receptor expressing Jurkat T cells could be activated by Axl+ tumor cells. Although not investigated in this study, we observed that the activation of Axl SynNotch is much stronger with plate-bound Axl than cells expressing Axl. We hypothesize that such discrepancy may be due to the suboptimal expression level of Axl on tumor cells. Furthermore, the surface stiffness (plastic vs. cell membrane) could also be a contributing factor since Notch receptor is known to be regulated by force^27–29^.

## Conclusion

Here we developed a humanized Axl scFv that can be exploited in the design of CAR and synNotch receptors. We validated the function of Axl CAR by demonstrating that human primary T cells expressing Axl CAR can effectively kill Axl+ tumor cells. We further showed the therapeutic potential of Axl scFv by designing a functional Axl synNotch receptor that can produce IL-10 when activated by Axl+ tumor cells. In further studies, Axl CAR and Axl synNotch expressing T cells can be tested in mouse xenograft studies to test *in vivo* efficacy.

## Methods

### Humanized single chain variable fragment (scFv) against Axl design

Humanized scFv against Axl was derived by fusing a variable region of an immunoglobulin heavy chain to the variable region of the light chain through a polypeptide linker (GS linker). Humanized heavy and light chain sequences were obtained from a previously published sequence.

### Axl CAR/synNotch construct design

Axl CAR was designed by fusing humanized Axl scFv to the hinge region of the human CD8α chain and transmembrane and cytoplasmic regions of the human CD28, 4–1BB, and CD3ζ signaling endodomains. They were under SFFV promoter for primary T cell experiments and under CAG promoter for Jurkat cell experiments. Axl synNotch receptor was designed by fusing humanized Axl scFv to the notch core intracellular domain fused to tTA transcription factor. Both Axl CAR and Axl synNotch contain a myc tag for verifying surface expression. Furthermore, the Axl CAR used in human primary T cell experiments was fused to a mCherry after the CD3ζ chain for expression level quantification. The Axl CAR used in Jurkat experiments was cloned into the PiggyBAC vector (System Bioscience Inc.), which has been modified by replacing the CMV promoter with a CAG promoter.

### Primary Human T cell Isolation and Culture

Whole peripheral blood was obtained from Boston Children’s hospital, as approved by the University Institutional Review Board (IRB) approved consent forms and protocols. Primary human CD8+ T cells were isolated from anonymous healthy donor blood by negative selection (STEMCELL Technologies #15063). T cells were cultured in human T cell medium consisting of X-Vivo 15 (Lonza), 5% Human AB serum (Valley Biomedical #HP1022), 10 mM N-acetyl L-Cysteine (Sigma-Aldrich #A9165), 55uM 2-mercaptoethanol (Thermo Scientific #31350010) supplemented with 50 units/mL IL-2 (NCI BRB Preclinical Repository). T cells were cryopreserved in 90% heat-inactivated FBS and 10% DMSO.

### Lentiviral Transduction of Human T cells

Replication-incomplete lentivirus was packaged via transfection of HEK 293 FT cells (Invitrogen) with a pHR transgene expression vector and the viral packaging plasmids: pMD2.G encoding for VSV-G pseudotyping coat protein (Addgene #12259), pDelta 8.74 (Addgene#22036), and pAdv (Promega). One day after transfection, viral supernatant was harvested every day for 3 days and replenished with pre-warmed Ultraculture media (Lonza #12-725F) supplemented with 2mM L-glutamine, 100U/ml penicillin, 100ug/mL streptomycin, 1 mM sodium pyruvate, and 50 mM sodium butyrate. Harvested virus was purified through ultracentrifugation or Lentivirus concentrator (Takara #631232). Primary T cells were thawed 2 days before ultracentrifugation and cultured in T cell medium described above. One day before ultracentrifugation, T cells were stimulated with Human T-activator CD3/CD28 Dynabeads (Thermo Scientific #11132D) at a 1:3 cell:bead ratio and cultured for 24 hr. After viral supernatant purification, rectronectin (Clontech #T100B) was used to transduce cells. Briefly, non-TC treated 6-well plates were coated with rectronectin following the supplier’s protocol. Concentrated viral supernatant was then added to each well and spun for 90 min at 1200xg. After centrifugation, viral supernatant was removed and 4 ml of human T cells at 250k/ml in T cell growth media supplemented with 100U/ml of IL-2 was added to well. Cells were spun at 1200xg for 60 min and moved to an incubator at 37 °C.

### Cancer Cell Lines

The cancer cell lines used were K562 myelogenous leukemia cells (ATCC # CCL-243) and Jurkat T cells. K562 and Jurkat T cells were cultured in RPMI-1640(Lonza#12-702Q) with 5% (v/v) heat-inactivated FBS, 2mM L-glutamine, 100U/ml penicillin and 100ug/mL streptomycin. Jurkat and K562 were electroporated or transfected with PiggyBac Transposon system (System Biosciences) to stably express Axl CAR, Axl synNotch receptor or surface antigen: AXL. Two days after transfection, antibiotic (Puromycin (Thermo Scientific #A1113803), zeocin (Thermo Scientific # R25005), or Hygromycin B (Thermo Fisher #10687010)) was added to the medium to select for cells that express the transgenes.

### T cell activation by plate bound antigen

Recombinant human Axl protein (R&D #154-AL-100) was coated on 96 well plate (flat bottom) overnight at 4 C°. Next day, the plate was washed with PBS three times to remove unbound Axl protein. Then, 200 × 10^3^ Jurkat T cells or human primary T cells engineered to express Axl CAR or Axl synNotch were added to each well. After 24 hr, for Axl CAR experiment, Jurkat or primary human CD8+ T cells were stained with α-CD69-APC (BD bioscience #340560) to measure CD69 expression level. GPF expression level driven form NFAT promoter was shown as NFAT promoter activity. For Axl synNotch experiment, BFP expression was measured as a tTA promoter reporter.

### Co-culture experiments

Jurkat T cells or primary T cells expressing Axl CAR (200 × 10^3^ cells/well/200ul) were incubated with K562 target cells (100 × 10^3^ cells/well) or with SK-OV-3 cells (100 × 10^3^ cells/well) at an E:T ratio of 2:1 or 1:1. For suspension cells, effector cells and target cells were mixed at the same time as seeding. For adherent cells (SK-OV-3), SK-OV-3 cells were pre-cultured for 12 hr. Then, Axl CAR T cells were added to wells. After 24 hr of co-culture, the supernatant was harvested and followed supplier’s protocol to determine IFN-γ, IL-2, IL-10 level. Cytokine release assays were carried out using IFN-γ, IL-2, or IL-10 ELISA Kit (BD Biosciences #555142, #555190, #555157). For detection of activation of T cell, expression CD69, GFP, and BFP were measured use Attune NxT flow cytometry (Thermo).

### Cytolysis assay

Two hundred thousand primary T cells expressing Axl CAR and control CD8 T cells were incubated with K562 target cells or SK-OV-3 cells (100 × 10^3^ cells/well). After 24 hr co-culture, the number of live K562 cells was counted by Attune NxT flow cytometry (Thermo). Live K562 cells were identified as 7-AAD^−^GFP^+^BFP^+^ cells. For detection of live SK-OV-3 after 24 hr co-culture, non-adherent cells were washed out, and adherent cells were stained with Calcein AM using LIVE/DEAD Viability/Cytotoxicity Kit (Thermo # L3224), following manufacturer’s protocol. For detection of cell-cell contact independent cytolysis activity, Axl-expressing K562 cells were incubated with conditioned medium from K562 cytolysis experiments described above. After 24 hr, the number of live K562 cells was counted by Attune NxT flow cytometry. For Jurkat cell targeting cytolysis assay, indicated number of Axl CAR-expressing primary T cells (0, 12.5 × 10^3^, 25 × 10^3^, 50 × 10^3^, 100 × 10^3^, 200 × 10^3^ cells) were cultured with 100 × 10^3^ Axl^+^ luciferase^+^ Jurkat cells for 4 hr. Culture medium was removed, and cells were resuspended with 50 ul/well of 2% FBS in PBS and lysed with 50 ul/well luciferin reagent (Promega #E2610). Lysates were transferred to 96-well plate (Corning #3904), and luminescence was measured with the SpectraMax M5 (Molecular Devices).

### Cell surface protein staining

After 24 hr stimulation, engineered Jurkat cells and primary T cells were stained with anti-CD69-APC antibody (BD bioscience #340560) for 30 min at room temperature. For detection of expression of cell surface synNotch, transfected Jurkat cells were stained with anti-Myc-PE (Santa Cruz Biotechnology, sc-40) for 30 min at room temperature. Fluorescence was measured by Attune NxT flow cytometry.

## Electronic supplementary material

Supplemental Information
