# Author Correction: Engineering Axl specific CAR and SynNotch receptor for cancer therapy

**DOI:** 10.1038/s41598-020-59902-7

**Published:** 2020-02-14

**Authors:** Jang Hwan Cho, Atsushi Okuma, Dalal Al-Rubaye, Ejaj Intisar, Richard P. Junghans, Wilson W. Wong

**Affiliations:** 1 Department of Biomedical Engineering, Boston University, Boston, MA 02215 USA; 2 Biological Design Center, Boston University, Boston, MA 02215 USA; 3 Biotechnology Department, College of Science, University of Baghdad, Baghdad, Iraq; 4 School of Medicine, Tufts University, Boston, MA 02111 USA

Correction to: *Scientific Reports* 10.1038/s41598-018-22252-6, published online 01 March 2018

The original version of this Article contained an error in Affiliation 3, which was incorrectly given as ‘Biotechnology Department, College of Science, Baghdad University, Baghdad, Iraq’. The correct affiliation is listed below:

Biotechnology Department, College of Science, University of Baghdad, Baghdad, Iraq

This error has now been corrected in the HTML and PDF version of the Article and in the accompanying Supplementary Information file.

