# Supplementary material for: Engineering Axl specific CAR and SynNotch receptor for cancer therapy
Source: Sci Rep. 2018 Mar 1;8:3846. doi: 10.1038/s41598-018-22252-6 (PMC5832765; doi:10.1038/s41598-018-22252-6)
Supplement: Supplementary file 1 — Supplemental Information [file 41598_2018_22252_MOESM1_ESM.docx]

**­Supplemental Information for**

**Engineering Axl specific CAR and SynNotch receptor for cancer therapy**

Jang Hwan Cho^1,2^^, Atsushi Okuma^1,2^^, Dalal Al-Rubaye^1,2,3^, Ejaj Intisar^1,2^, Richard P. Junghans^4^, and Wilson W. Wong^1,2*^

^1^Department of Biomedical Engineering, Boston University, Boston, MA 02215, USA.

^2^Biological Design Center, Boston University, Boston, MA 02215, USA.

^3^Biotechnology Department, College of Science, University of Baghdad, Baghdad, Iraq.

^4^School of Medicine, Tufts University, Boston, MA 02111

^These authors contributed equally.

*Correspondence: [wilwong@bu.edu](mailto:wilwong@bu.edu).


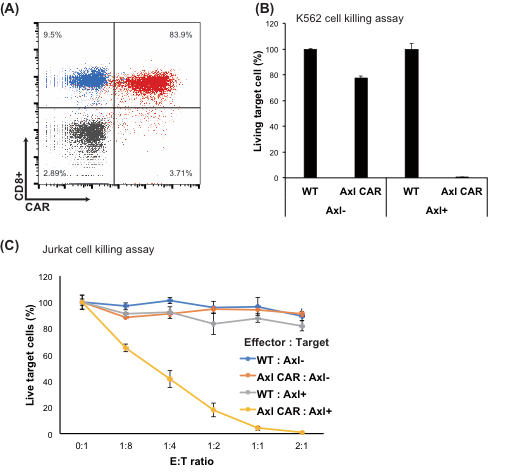
 **Supplementary Fig.1 Human primary CD8 T cells expressing Axl-CAR show effective *in vitro* cytotoxicity against Axl+ target cancer cells.**

(A) Axl CAR expression level in human primary CD8+ T cells. (B) Killing assay against K562. Live K562 cells were counted by flow cytometry analysis. Graph indicates the ratio to live K562 cells in the condition of co-culture with WT T cells. (C) Killing assay against Jurkat cells. Axl+ and Axl+ Jurkat cells express luciferase. Graph indicates the percentage to the luminescence in no effector T cell condition. Co-culture was performed in the indicated ratio of effector and target (E:T) for 4 hr. Data are representative of three biological replicates and presented as the mean ± SD.
